# The Role of Diet, Glycaemic Index and Glucose Control in Polycystic Ovary Syndrome (PCOS) Management and Mechanisms of Progression

**DOI:** 10.1007/s13668-024-00601-4

**Published:** 2025-01-03

**Authors:** Claire Johnson, Gökçen Garipoğlu, Yvonne Jeanes, Giada Frontino, Adele Costabile

**Affiliations:** 1 School of Life and Health Sciences, University of Roehampton, London, UK; 2 Faculty of Health Sciences, Department of Nutrition and Dietetics, Bahçeşehir University, Istanbul, Turkey; 3 Consultant Obstetrician and Gynaecologist, London, England

**Keywords:** Polycystic Ovary Syndrome; Endocrine Disorder, Low Glycaemic Index Diet, Glucose Tolerance, Impaired Glucose Tolerance

## Abstract

**Purpose of Review:**

Polycystic Ovary Syndrome (PCOS) is a complex endocrine disorder with several causal pathways including impaired glucose tolerance, insulin resistance (IR), compensatory hyperinsulinemia and excess androgens (hyperandrogenism). This heterogeneous condition causes a range of reproductive, metabolic and psychological implications, the severity of which can differ between individuals depending on factors such as age, diet, ethnicity, genetics, medication, contraceptive use, adiposity, and Body Mass Index (BMI).

**Recent Findings:**

Dietary interventions that focus on a low glycaemic index and glucose control are an efficient first-line dietary solution for the management of impaired glucose tolerance and IR, which subsequently improves weight management, quality of life and PCOS-related symptoms in individuals with this condition.

**Summary:**

This review aims to explore the relevance of nutrition and more specifically, the association of glycaemic index and glycaemic load with PCOS, as well as to assess the potential benefits of manipulating those indexes in the dietary approach for this syndrome.

## Introduction

Polycystic ovary syndrome (PCOS) is a complex yet common endocrine disorder affecting around 5–20% of reproductive aged women, depending on the diagnostic criteria used and population studied [1]. PCOS is a heterogeneous condition which can cause a diverse range of reproductive, metabolic and psychological implications, the severity of which may differ between individuals depending on factors such as age, diet, ethnicity, genetics, medication, contraceptive use, adiposity, BMI and geographical region [2]. The heterogeneous nature of PCOS complicates its pathophysiology. However, several key causal mechanisms associated with the exacerbation of PCOS have been identified. These are insulin resistance (IR), compensatory hyperinsulinemia, excess androgens and male-predominant hormones (hyperandrogenism) and impaired glucose tolerance (IGT). A reported 95% of obese and 75% of lean women with PCOS have IR [3] resulting in a 2–fourfold higher rate of metabolic syndrome in women with PCOS compared with the general population [4]. Despite the high prevalence rate of IR in lean women with PCOS, weight loss remains the most commonly reported clinical recommendation for PCOS management [5].

In addition, environmental and genetic factors may contribute to its aetiology, further adding to this multi-system syndrome [6]. These complexities, together with high variability and conflicting results across studies, have led to a lack of clear long-term solutions.

## Purpose of Review

Diet can significantly influence IR and glucose tolerance in individuals with PCOS. While there are many potential complex hormonal imbalances and interactions in PCOS, this review will discuss the prevalence and role of IR, impaired glucose tolerance, and hyperandrogenism in women with PCOS. It will explore differences in PCOS phenotypes (including lean versus obese body types) and how these impact clinical diagnosis, symptoms and long-term dietary management. It is important that dietary interventions and supplementation are relevant for all PCOS types, beyond just weight loss. A better understanding of IR and IGT are crucial for providing effective clinical guidelines and individualised dietary management solutions for PCOS, which thus far appear to be lacking.

## Diagnostic Criteria

There are several widely used diagnostic criteria for PCOS, including the 1990 NIH Criteria, the 2003 Rotterdam Criteria, and the 2006 Androgen Excess-PCOS criteria [7]. According to the most commonly used Rotterdam criteria (endorsed by the International Evidence Based Guidelines the Assessment and Management of PCOS), PCOS is diagnosed when at least two of the following are met by patients: (i) clinical or biochemical hyperandrogenism, (ii) anovulation or menstrual cycle irregularity, (iii) polycystic ovaries by ultrasound [8]. In this sense, a woman may have polycystic ovaries without PCOS which must involve clinical or biochemical hyperandrogenism and/or menstrual irregularity. PCOS is a syndrome, meaning that associated symptoms may occur independently or together with differing severity in each individual. The severity of symptoms may be dependent on PCOS phenotype; (type A) polycystic ovaries, chronic anovulation and hyperandrogenism; (type B) hyperandrogenism and chronic anovulation; (type C) hyperandrogenism and polycystic ovaries; (type D) chronic anovulation and polycystic ovaries [9]. These phenotypes are associated with different metabolic risks [8].

However, differing diagnostic criteria, definitions and measurement methods related to biochemical markers has led to high rates of underdiagnosis, misdiagnosis and overdiagnosis of PCOS, as well as substantial heterogeneity across research, contributing not just to patient anxiety, but confusion regarding management and intervention [10]. In fact, a comprehensive international investigation including 1385 women found that only 15.6% were satisfied and understood the information received at the time of diagnosis regarding PCOS and its management [11]. In addition, a review by Nemchikova and Frontoni [5] found that clinical advice was focused largely on weight loss without acknowledgement of individual body composition or PCOS type, highlighting the need for better understanding of PCOS phenotypes.

## PCOS Phenotypes

PCOS phenotypes distinguish and acknowledge in individual body types (lean, overweight and obese) as well as which causal mechanisms are most predominant in each woman with PCOS. Increased body fat has been associated with more severe metabolic and reproductive consequences such as hyperandrogenism, dyslipidaemia and IR in women with PCOS [12]. In addition, PCOS symptom severity, IR, increased BMI and adverse metabolic outcomes are significantly associated with phenotype A (polycystic ovaries, anovulation and hyperandrogenism), whereas women with phenotype D (polycystic ovaries and anovulation) have been found metabolically similar to non-PCOS controls with markedly less IR [13, 14]. However, there is no universally accepted definition of metabolically healthy obesity (MHO). Most studies define MHO as having either 0, 1, or 2 metabolic syndrome components, whereas many others define MHO using the homeostasis model assessment of insulin resistance (HOMA-IR). Therefore, numerous people reported as having MHO are not metabolically healthy, but simply have fewer metabolic abnormalities than those with metabolically unhealthy obesity (MUO). Nonetheless, a small subset of people with obesity has a normal HOMA-IR and no metabolic syndrome components. People who are reported as having MHO are often not truly healthy, but simply have fewer cardiometabolic abnormalities than those defined as MUO. The data from longitudinal studies suggest that approximately 30% to 50% of people with MHO convert to MUO after 4 to 20 years of follow-up. Very few people with obesity are truly metabolically healthy.

The studies to date have not demonstrated important differences in lifestyle factors (diet composition, physical activity, and sleep) between MHO and MUO. However, this does not mean that lifestyle is not an important regulator of metabolic health, but rather underscores the limitations in the assessment of lifestyle factors and in the definition of MHO in the current studies. The mechanism(s) responsible for the divergent effects of obesity on metabolic health is not clear, but studies conducted in rodent models suggest that differences in adipose tissue biology in response to weight gain can cause or prevent systemic metabolic dysfunction. However, even without overweight and obesity women with PCOS still present with increased prevalence of glucose metabolic disturbances, IR and increased metabolic risks such as cardiovascular disease compared to BMI-matched controls [15]. Clinical advice and long-term management for women with PCOS would therefore benefit from further investigation and acknowledgement of PCOS phenotypes. Solutions might benefit from looking beyond the commonly prescribed ‘weight loss’, and instead focus on investigating the presence of IR across all PCOS individuals and types.

## Insulin Resistance (IR)

Despite decades of research highlighting the mechanistic role of IR in up to 80% of women with PCOS [9, 14, 16, 17], IR is not part of any diagnostic criteria. This may be attributable to a lack of accessibility of accurate measurement methods for IR leading to conflicting results across research [17]. Nonetheless, the mechanistic action of IR in PCOS is important for current understandings of this syndrome.

**Fig. 1:**
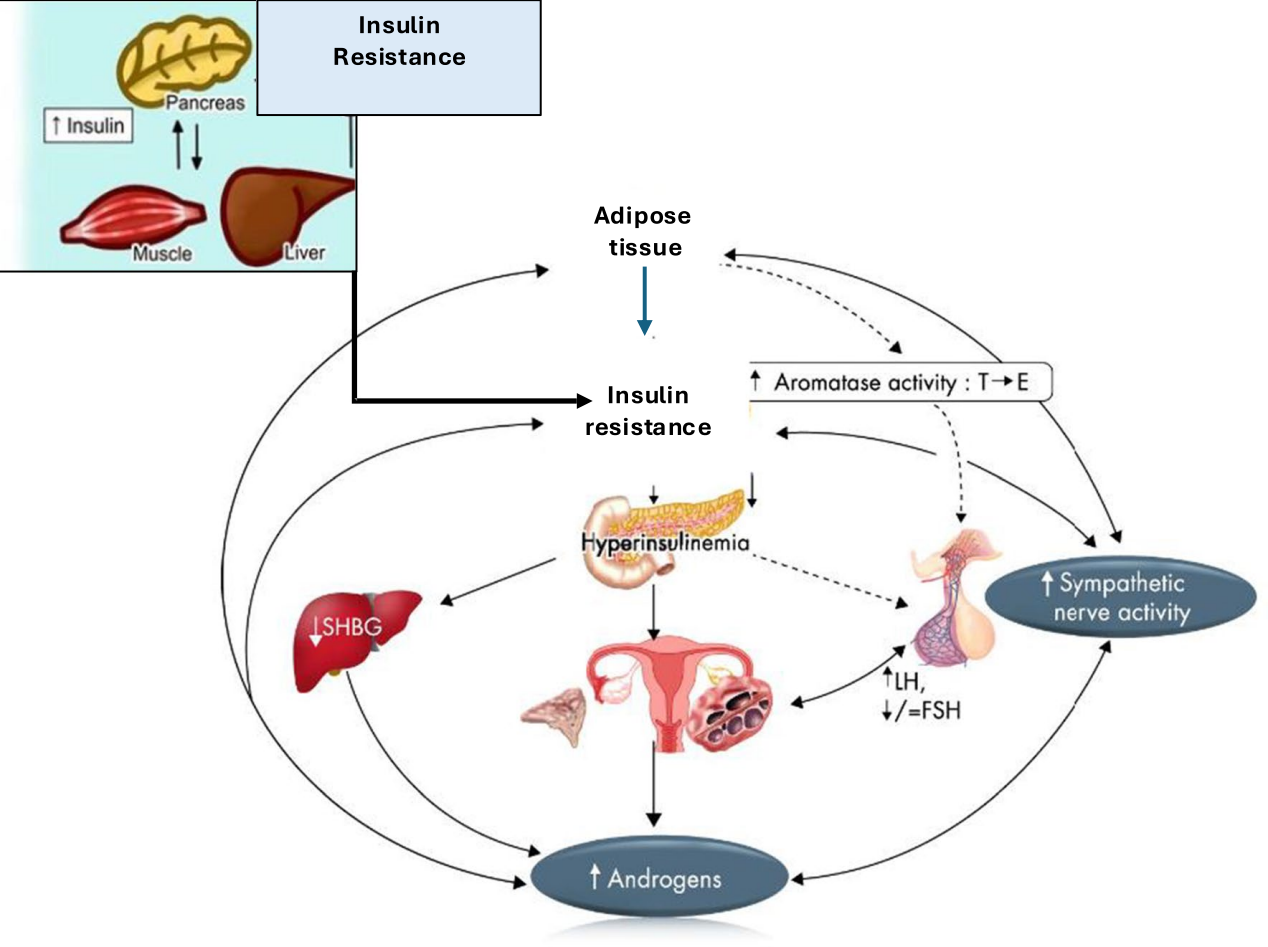
Pathophysiology of PCOS: a vicious circle [modified by [24]

In a typical woman, insulin acts directly on the ovaries via the stimulation of thecal cells, which in turn stimulates normal levels of androgen production [18]. In individuals where IR is present, abnormal function and signalling of insulin receptors leads to compensatory hyperinsulinemia [3]. In response to hyperinsulinemia ovarian theca cells which normally support growing follicles and the generation of mature oocytes become hyper-responsive to excess insulin, causing them to proliferate and subsequently increase further androgen secretion [19]. Hyperinsulinemia further aggravates PCOS by inhibiting the production of insulin-like growth factor (IGF-1) binding protein in the liver, which causes elevated circulating IGF-1 levels, in turn stimulating more ovarian thecal cell androgen production, as well as reducing the production of sex-hormone-binding globulin (SHBG), thereby leading to increased free testosterone levels, further exacerbating symptoms of hyperandrogenism [20]. The resulting combination of hyperinsulinemia and hyperandrogenism disrupts follicle growth, underpinning some of the most common symptoms associated with PCOS; anovulation, menstrual irregularity, hirsutism, acne, male pattern balding and weight gain. Hyperinsulinemia and hyperandrogenism appear to exist bidirectionally, as both conditions can influence and exacerbate each other PCOS, however their interactions and ‘which one comes first?’ is a question still not clearly understood (Fig. 1). Although IR appears to be the most prominent mechanism driving PCOS, genetics, adrenal dysfunction, thyroid dysfunction and inflammation are other potential pathways which may contribute to hyperandrogenism and PCOS symptoms [6, 7] therefore hyperandrogenism is not always indicative of IR (and vice versa).

Since visceral fat and excess adipose tissue play a key role in generating and exacerbating an insulin-resistant state [15, 20, 21], a 5% reduction in weight is often the first line of recommendation for PCOS women [21]. However, the presence of IR in lean women with PCOS has led to the concept of intrinsic IR, unrelated to body composition and weight. A meta-analysis by Shang et al. [3] including 1193 participants across 19 trials found that 75% lean women and 95% of obese women with PCOS had IR. A robust systematic review and meta-analysis by Cassar et al. [22] found that women with PCOS were 27% less insulin sensitive than controls, independent of BMI, age, diagnostic criteria and ethnicity, concluding that PCOS is underpinned by an intrinsic IR. However, BMI independently exacerbated IR by 15% in PCOS. Additionally, one of the first controlled trials studying IR and glucose tolerance in women with PCOS by Legro et al. [23] found that both lean and obese women with PCOS are at significantly increased risk of IR, IGT and type 2 diabetes (T2D) compared to concurrently studied age, weight and ethnicity comparable women without PCOS.

These findings demonstrate the need for solutions above and beyond weight loss target intrinsic IR and IGT, which are likely to differ mechanistically from extrinsic IR related to body composition and weight.

## Treatment and Management: Diet and Supplementation

Weight management, along with lifestyle changes such as diet, physical activity, and behaviour modification, is considered the primary treatment approach in international guidelines for PCOS. Although these guidelines advise following general dietary and physical activity recommendations for the population, there is growing interest and ongoing research into the potential benefits of integrating psychological support and sleep improvement strategies. Additionally, various traditional, complementary, and integrative medicine (TCIM) therapies are being explored for their role in optimizing PCOS management. Currently, there is insufficient evidence to support a specific dietary composition for PCOS, as adjustments in protein, carbohydrate, or fat intake generally result in similar outcomes in addressing PCOS symptoms [25].

## The D-A-S-H diet and the Mediterranean Diet (MD)

Taking the above findings into account, it is widely agreed that reducing IR and improving glycaemic control has great benefits for the metabolic and hormonal state of women with PCOS [26]. While weight loss may not be relevant for lean women with PCOS and in targeting intrinsic IR, reducing intake of high glycaemic index (GI) carbohydrates offers promising results for all PCOS body types and phenotypes [6]. The concept of a low-glycaemic index (GI) diet aims to reduce glycaemic load (GL) and therefore reduce subsequent blood sugar spikes and insulin responses to ingested foods and drinks, contributing to reduced IR [27].

Dietary interventions have been strongly linked to improvements in PCOS symptoms such as menstrual regularity, spontaneous ovulation, insulin sensitivity and weight loss [9]. A meta-analysis and systematic review by Shang et al. [3] including 1193 participants found that diet was more effective than exercise and the commonly prescribed insulin-sensitising drug, metformin, for women with PCOS across metabolic and reproductive parameters. In particular, the DASH diet (Dietary Approaches to Stop Hypertension) based on 50–55% (total daily energy intake) from whole grain carbohydrates, plus encouraged high intake of fruit, vegetables, nuts, legumes and low-no intake of sodium, saturated fat meat, sugar and alcohol was an effective dietary pattern for improving insulin sensitivity measured using HOMA-IR scores, especially when compared to lower carbohydrate diets (30–40% total daily intake). The MD, similar in nature to the DASH diet but with fewer absolute restrictions, was also strongly associated with reduced IR and better glycaemic control compared to other diets. However, calorie-restricted diets (reducing calories by 500 kcal/d) were more advantageous for weight loss compared with DASH, low-carbohydrate and the MD. Another study found negative correlations between the degree of adherence to the MD and the clinical severity of PCOS, where lower intake of fibre, fruit, vegetables and monounsaturated fat contributed to more severe IR and hyperandrogenism [28]. The latest work of Barrea and collaborators [29] have highlighted the interdisciplinary collaboration between endocrinologists and nutritionists for the optimal management of endocrine disorders, including the potential role of MD in their prevention and management.

## The Role of Fibre and Glycaemic Index (GI)

Large-sample control trials and prospective studies assessing the impact of the DASH and Mediterranean diet on PCOS outcomes is sparse, however there is an overwhelming amount of evidence across other population groups supporting the positive role of high fibre intake, fruit and vegetables and low glycaemic index carbohydrates on metabolic health, inflammation, gut health, body composition, satiation and adherence [30].

High fibre intake in particular helps to reduce the GI and glycaemic load of food and meals, which leads to reduced blood sugar spikes and therefore less circulating insulin and subsequent IR [31]. Soluble fibre specifically has been found to be most effective in controlling glycaemic responses and IR [32]. Increased fibre intake was found to be a strong predictor of decreased BMI in 57 women with overweight and PCOS (BMI > 27 kg/m^2^) aged between 18–40 years over a 16-week duration [12]. In addition, fibre’s ability to add bulk to food whilst maintaining glycaemic control may importantly lead to satiation and appetite control.

A trial by Marsh et al. [13] found that 29 women with overweight and PCOS (mean BMI = 34 kg/m^2^, mean age 31 years) assigned to a low GI diet lost more weight and gained menstrual cycle regularity compared with 20 women with overweight and PCOS (mean BMI = 34 kg/m^2^, mean age 29) assigned to a conventional healthy diet. Both diets were matched in energy and composition except for the quality of carbohydrate (i.e. GI) varying between the two. Although most participants across both groups failed to reach a 7% weight loss goal set in the trial, the low GI diet still provided a threefold greater improvement in whole-body insulin sensitivity measured via responses to a 75 g OGTT, as well as overall increased weight loss. In addition, 95% of women in the low-GI diet group reported menstrual cycle regularity and ovulation vs 63% in the conventional healthy diet group, all of whom reported menstrual cycle irregularity at baseline. The mechanisms thought to underpin these improvements in menstrual cycle regularity and ovulation is linked to improvements in insulin sensitivity through weight loss. However, a key limitation in this study was the inclusion of women taking the insulin-sensitising drug, Metformin, which increases insulin sensitivity.

Research into other IR-driven metabolic disorders such as T2D can offer additional valuable insight, especially since the prevalence of T2D has been found to be up to 10 times higher in women with PCOS compared to healthy control women [33]. A large-scale multi-country systematic review and meta-analysis including 10,000 adults with IR related to T2D found that high fibre intake of 35 g per day compared to 19 g per day resulted in significant reduction of fasting plasma glucose, IR and body weight [34]. In addition, new insights from an 8 yr UK general practice evaluation on low-carbohydrate diets and weight loss reported an average 10 kg weight loss in 186 patients with T2D over an average of 33 months, where the reduction of HbA1c levels reversed T2D in 51% of the cohort [17]. Although this study was not PCOS-specific and included varying age groups across males and females, the mechanisms by which a low-carbohydrate diet can lower HbA1c levels, IR and cause weight loss are relevant.

## Very Low Carbohydrate Diets (VLCD) and Keto Diets (KD)

Very low carbohydrate diets (VLCD), keto diets (KD) and energy restricted diets may have clinical relevance for women with overweight or obesity and PCOS due to their ability to induce short-term weight loss and improve body composition. Strong clinical evidence links excess adipose tissue with metabolic and hormonal disruptions, leading to and exacerbating IR and impaired glucose control [35]. Obesity reduces the levels of metabolically favourable hormones associated with insulin sensitivity such as adiponectin [12, 36]. The downregulation of adiponectin is linked to IR and inflammation, with visceral fat being a key player in generating an insulin-resistant state leading to impaired glucose tolerance (IGT) [37]. In this sense, the reduction of adipose tissue may be the most important goal for women with overweight or obesity and PCOS to restore metabolic and reproductive functions [38]. A meta-analysis by Zhang et al. [21] including 327 participants found that low-carbohydrate diets (< 45% of total daily energy intake) significantly reduced BMI in women with PCOS as well as significantly increasing Sex-Hormone-Binding-Globulin (SHBG), thereby reducing hyperandrogenism. Low-carbohydrate, low fat diets were associated with decreased levels of circulating insulin, glucose and inflammatory markers which play a critical role in metabolic homeostasis, ovarian function and reduced androgens.

The KD which restricts carbohydrate intake to 5–10% of total daily energy intake is another well studied dietary intervention in the general context of weight loss [27]. A 12-week trial by Paoli et al. [39]. with 14 women with overweight and PCOS revealed significant BMI, body fat mass and visceral fat reduction as well as reductions in glucose, insulin blood levels, free testosterone and DHEAS after following a ketogenic diet of 1600–1700 kcal per day. However, the practicality, satiety, enjoyment and social aspect of the KD must be considered to provide realistic long-term solutions. The restrictions involved in KD may make adherence difficult and limit practical application, plus it is widely accepted that KDs are not recommended for long term use [39].

## The Impact of Diet on Glucose Tolerance and Insulin Sensitivity

Glucose tolerance and insulin sensitivity are clearly relevant metabolic states for better reproductive outcomes in all women with PCOS, regardless of body composition [40]. KD and energy restricted diets may be useful short-term to induce weight loss in women with overweight or obesity and PCOS, whereas Mediterranean style dietary patterns with a focus on high fibre, low GI carbs, may be beneficial for targeting intrinsic IR. Long term, the Mediterranean-style dietary patterns may be beneficial for all PCOS women due to its positive impact on IR, glucose tolerance, inflammation, gut health, satiation and adherence levels, plus reduced intake of SFAs and refined carbohydrates, and high intake of antioxidants, monounsaturated fats and additional vitamins and minerals may offer additional benefit to women with PCOS [41].

Further investigation into exact dietary macronutrient composition for PCOS management is required.

## PCOS and Gut Microbiome

A novel concept of "microgenderome" has recently been introduced in relation to the potential bidirectional interaction functions between the intestinal microbiota and sex hormones. It has been reported that intestinal microbiota differs between the sexes during adolescence and especially influences testosterone hormone level [42–45]. Excessive androgen production and insufficient oestradiol are typical of women with PCOS. Such hormone changes in PCOS are probably related to dysbiosis of the gut microbiota [42]. Research has indicated that the gut microbiota of individuals with PCOS is associated with the onset and progression of IR, hyperandrogenism, chronic inflammation, and metabolic syndrome. Additionally, the gut microbiota may impact the clinical manifestations of PCOS via the brain-gut axis, sex hormones, lipopolysaccharide, and short chain fatty acids (SCFAs). [43]. Significantly, butyrate (a type of SCFAs) has demonstrated the ability to modulate the production of P4 (progesterone) and E2 (estradiol) in porcine granulosa cells (PGCs) through the cAMP signalling pathway [44].

A few studies showed that, women with obesity and PCOS have elevated enterobacteria, decreased lactobacilli and bifidobacteria, and alterations in gut microbiota are associated with inflammatory levels and IR, in contrast to lean women with PCOS and healthy controls [45]. Moreover, variations exist in the composition and organization of gut microbiota among women with PCOS with and without IR and found that the abundance of *Prevotella* decreased and *Bacteroides* increased in the former group [42]. It is hypothesized that the rise of *Lactobacillus* spp. in the gut enhances the generation of SCFAs, improves the barrier function of the gut and lowers the transfer of bacterial endotoxins along the intestinal wall where they might generate inflammation and IR [46]. To test the potential impact of *Lactobacillus* spp. on IR, rats with PCOS were treated with Faecal microbiota transplantation (FMT) and *Lactobacillus*’ transplantation in an experimental study. The findings demonstrated that after receiving FMT and Lactobacillus ‘transplantation for PCOS rats, serum testosterone levels decreased, estrous cycles improved and ovarian function normalised [42].

These studies emphasise the need for more careful evaluation of the effect of dietary components on microbiota dysbiosis in women with PCOS and the importance of dietary interventions. Especially in recent years, the effects of ketogenic diet (KD) applied for weight loss in the presence of obesity and IR on gut microbiota in women with PCOS need to be further investigated [47, 48]. PCOS mice subjected to a KD for 8 weeks had reduced body weight increase, diminished luteinizing hormone and androgen levels, and enhanced insulin levels. Moreover, KD rectified the dysregulation of gut microbiota by modulating the ratio of Firmicutes and Bacteroidetes. However, this study found contradictory effects of KD on the gut microbiome in PCOS [48]. Overall, the large body of studies demonstrated that gut microbiota could regulate the synthesis and secretion of insulin, and affect androgen metabolism and follicle development, providing us a novel idea for unravelling the pathogenesis of PCOS.

## Supplementation

### Inositol

The important role of improved insulin sensitivity and glucose control in PCOS can be demonstrated by the efficacy of insulin sensitising supplements in PCOS population groups. In conjunction with lower glycaemic index diets, insulin-sensitising drugs appear to reduce the severity of PCOS symptoms and restore ovulatory function. The most prescribed insulin-sensitising drug is Metformin with well-researched benefits regarding its role in improving fertility. However, patient compliance is often affected by side effects e.g. nausea and diarrhoea and potential risk of foetal abnormality [49]. As a result, substantial research has looked towards other insulin-sensitising drugs without side effects. Myo-inositol (MI) and di-chiro-inositol (DCI) are two stereoisomers of inositol which may have a key therapeutic role in PCOS due to their insulin sensitivity modulatory action. Inositol supplementation has been found to reduce ovulatory dysfunction, IR, hyperinsulinemia and hyperandrogenism, thereby improving fertility and reproductive outcomes with fewer side effects at a lower cost [50]. Large-sample studies comparing the therapeutic use of metformin vs inositol in PCOS management are sparse, however one study found that MI plus 400 μg folic acid taken orally over 6 months restored spontaneous ovulation in 65% of patients compared to 50% of patients taking 1500 mg/day metformin [49]. A systematic review and meta-analysis of 26 RCTs by Greff et al. [1] found that inositol supplementation was non-inferior when compared to Metformin. The use of inositol for PCOS has been researched for decades. Weight management A trial by Nestler et al. [50] using a daily dose of 1.2 g of DCI inositol in the treatment of 22 hyperinsulinemic women with PCOS found that 19 out of 22 participants restored ovulation compared to the placebo group and improved their hormonal profile. Other studies have shown that 4 g daily dose of MI improves hormonal profile, restores ovulation and is able to induce menstrual cycle regularity in both lean women with PCOS and women with obesity and PCOS [51]. In agreement, a meta-analysis by Pundir et al. [52] including 601 women with PCOS found that treatment with inositol (either myo-inositol, di-chiro inositol or both together) significantly increased ovulation rate, as well as frequency of menstrual cycles sixfold without negative side effects. Consistent improvement in glycaemic parameters (such as fasting glucose, insulin levels, IR) were also observed in women taking inositol compared to those receiving the placebo comparator. In addition, the meta-analysis also found levels of total androgens, serum testosterone and dehydroepiandrosterone (DHEAs) decreased whilst SHBG levels were improved.

While both MI and DCI monotherapies have beneficial effects on ovulation and insulin sensitivity in women with PCOS, the role of combined treatment of MI plus DCI has been investigated and continues to offer a promising solution. In particular, a ratio of 40:1 MI:DCI has been shown to improve ovarian function as well as hormonal and metabolic state in women with PCOS more efficiently than either MI or DCI alone [50]. It is well supported that mechanisms of MI improve ovulatory function while mechanisms of DCI improve glycaemic parameters and reduce hyperinsulinemia [49]. Based on this mounting evidence, the International Consensus Conference on MI and DCI in Obstetrics and Gynecology reviewed seminal experimental papers and randomised clinical trials on the role inositol in clinical practice. They recommended the treatment of PCOS using a combination of both molecules in the ratio of 40 (MI): 1(DCI) [51].

### Berberine

Emerging research investigating the insulin-sensitising effects of Berberine is also showing small but promising results. Berberine is an isoquinoline alkaloid, commonly used botanicals, such as *Coptis chinensis Franch* [53]. Berberine has been found to lower blood glucose, improve blood lipid regulation and modulate antibacterial activity. The mechanisms underpinning these improvements appear to be in berberine’s ability to promote glucose transport and consumption and lipid metabolism, positively impacting lipid synthesis, anti-inflammatory cytokine secretion, oxidative stress reduction and shaping the gut microbiota by significantly reducing the Firmicutes and Bacteroides proportion [54]. Berberine could therefore serve as an important adjuvant in the medical treatment of PCOS, however it is not yet recognised in clinical settings as further studies into the use and efficacy of berberine in PCOS are needed.

### Carnitine

Carnitine, derived from the amino acids lysine and methionine, plays a crucial role in glucose and fatty acid metabolism, particularly in its active form, L-carnitine [55] Research has found that some women with PCOS tend to have lower L-carnitine levels, which is linked to poor oocyte quality, IR, and increased androgens [56]. Carnitine deficiency may disrupt mitochondrial function and affect insulin signalling. A study of 80 women with PCOS found that 3 g of L-carnitine daily for three months improved insulin sensitivity, BMI, and LDL levels [57], however research into L-carnitine, insulin sensitivity and PCOS are sparse.

Other supplementations and individual food substances of interest within PCOS research include CoEnzyme Q10, N-Acetyl Cysteine, curcumin, flaxseed and cinnamon. For instance, a study with IR rats found that COEnyzmeQ10 improved markers of insulin sensitivity through modulation of insulin, glucose uptake, and adiponectin receptors and reduction of ROS concentrations [58]. Food substances and herbs such as curcumin, cinnamon and flaxseed show small but promising results for supporting glucose tolerance and insulin sensitivity. For example, a review of supplementation in PCOS found that curcumin may exert hypoglycaemic effects through attenuating TNF-α concentrations and plasma free fatty acids. Cinnamon may exert hypoglycaemic effects through insulin receptor autophosphorylation and dephosphorylation; GLUT-4 receptor synthesis and translocation. The lignans and fibre in flaxseed may improve insulin sensitivity by reducing glucose uptake speed and insulin release, and ω−3 fatty acids may increase adiponectin concentrations [59]. However, the vast diversity and heterogeneity of interventions, treatment regimens, doses and consistency in supplement and herbal formulations across research makes interpretation difficult and firm conclusions cannot yet be reached. Whilst mechanisms of insulin sensitising supplements are clearly a crucial part of the wider PCOS picture, further robust evidence is required to integrate these solutions into clinical use.

Whilst the heterogeneity of research around supplements, foods and herbs in PCOS make conclusions hard to draw, substantial research clearly demonstrates the benefits of inositol supplementation in women with PCOS, especially in improving ovulation rates, menstrual cycle regularity and glycaemic parameters. Recent recommendations from the International Evidence-based Guideline for the Assessment and Management of PCOS state that inositol (in any form) should be considered an experimental therapy in PCOS, highlighting the importance of further research [29].

## Conclusions and Future Perspectives

IR and IGT are clinically relevant states in women with PCOS, where improvements can significantly reduce PCOS symptom severity. IR, whether intrinsic or extrinsic, and its interrelation with hyperandrogenism are important mechanisms involved in PCOS and its symptoms, however the severity of PCOS might depend on phenotype, weight, body composition, ethnicity, age, genetics, medication and oral contraceptive use. Moreover, determining the phenotype of PCOS during its diagnosis will be helpful for better management of PCOS but surely more thorough investigations are recommended in this regard. Large amounts of heterogeneity across studies caused by these co-founding factors have led to much conflicting and inconclusive evidence, consequently leading to a lack of clear PCOS management recommendations and clinical guidance.

Lifestyle or diet, genetics, gut dysbiosis, neuroendocrine alterations, and obesity are among the risk factors that predispose females to PCOS. Dysbiosis of gut microbiota may play a pathogenic role in the development of PCOS. Research into dietary interventions and supplementation offers promising new solutions for PCOS management. Low-carbohydrate and energy restricted diets, plus inositol supplementation may support women with overweight or obesity and PCOS reach the recommended 5% reduction in weight loss, however this is not relevant to all women with PCOS and is often only a short-term solution. The existence of intrinsic IR and increased metabolic risks in lean women with PCOS demonstrates the need for better understanding of these mechanisms to provide relevant targeted solutions beyond weight loss. Reductions in IR and IGT appear to be effective and beneficial in all PCOS types, which can be achieved efficiently through nutrient-dense diets focused on low GI-high fibre carbohydrates as well as inositol supplementation, specifically MI and DCI in a ratio of 40:1. The reduction of IR and IGT have been shown to play a fundamental role in restoring hormonal balance, menstrual cycle regularity, ovulation and fertility, as well as reducing negative symptoms associated with hyperandrogenism and decreasing metabolic risks. Ongoing research into the genetic and physiological aspects of PCOS is critical for discovering both preventive measures and effective treatments. Further investigation is needed to determine whether changes in gut microbiota composition result from steroid alterations in individuals with PCOS and to uncover the mechanisms behind these changes. Additional studies are necessary to evaluate the role of these supplements in PCOS treatment or prevention. Randomized clinical trials are essential to clarify the link between gut microbiota imbalance and PCOS. Comprehensive future studies may enable the use of gut microbiota as a biomarker and lead to personalized microbiota-targeted therapies for PCOS. Current treatments focus on managing symptoms rather than addressing the underlying condition itself. Therefore, in-depth research is needed to enhance treatment options and mitigate the long-term health consequences of PCOS.

## Data Availability

No datasets were generated or analysed during the current study.
